# Cardiac arrest after thoracic paravertebral block with ropivacaine in a 6-year-old child

**DOI:** 10.1186/s40981-015-0015-4

**Published:** 2015-12-29

**Authors:** Yu Yamane, Tetsuro Kagawa

**Affiliations:** Department of Anesthesiology, Hyogo Prefectural Kobe Children’s Hospital, 1-1-1, Takakuradai, Suma-ku, Kobe, Hyogo 654-0081 Japan

**Keywords:** Local anesthetic systemic toxicity, Thoracic paravertebral block, Cardiac arrest, Ropivacaine, Child

## Abstract

We encountered cardiac arrest induced by 0.375 % ropivacaine 7 ml administered via a catheter for continuous thoracic paravertebral block (TPVB) in a 6-year-old female who underwent pacemaker implantation for sick sinus syndrome (SSS). She was successfully resuscitated with adrenaline and lipid emulsion. Plasma concentration of ropivacaine was 5.2 μg/ml, suggesting intravascular administration of ropivacaine. Inadvertent intravascular administration is a crucial complication of TPVB.

## Background

TPVB is effective for intra- and postoperative analgesia for thoracotomy and laparotomy in children as well as in adults [1, 2]. Although it has been considered to be safer than epidural anesthesia from the point of epidural hematoma and spinal cord injury, as well as being able to be performed with ultrasound guidance, rare complications such as vascular puncture may occur [3]. We report a case of cardiac arrest in a child induced by ropivacaine administered via an indwelling catheter for TPVB.

## Case presentation

A 6-year-old, 14 kg, 91 cm girl with trisomy 21 was scheduled for elective pacemaker implantation with lead attachment to the epicardium under thoracotomy. She was born with trisomy 21, and presented with coarctation of aorta and atrioventricular septal defect, which have being surgically repaired at the age of 9 days and 9 months, respectively. SSS as well as bradycardia-tachycardia syndrome was diagnosed by manifestation of bradycardia and tachycardia after the surgical repair of mitral insufficiency at the age of 3 years. Her pulse control was unfavorable despite the administration of isoprenaline hydrochloride, leading to a decision of pacemaker implantation. Diazepam (9 mg) was given as a premedication. Anesthesia was induced uneventfully with sevoflurane (5 %) in 66 % nitrous oxide and 33 % oxygen although we prepared atropine and transcutaneous pacing in case of hemodynamic deterioration. After tracheal intubation, anesthesia was maintained with propofol (10 to 12 mg/kg/h) and remifentanil (0.1 to 0.2 μg/kg/min). With anesthesia induction, the heart rate decreased from approximately 140/min (atrial tachycardia) to 120/min, changing from atrial tachycardia to atrial flutter, but the blood pressure was maintained. The ultrasound-guided TPVB was performed through the 5th intercostal space, with the patient placed in the right lateral position. A probe was placed in parallel to the costal bone, and an 18G Tuohy needle was inserted from the lateral side using the in-plane approach. When the needle tip reached the paravertebral space, 5 mL of saline was infused to confirm the ventral transposition of the pleura and widening of the paravertebral space. Subsequently, a catheter was inserted into the paravertebral space (length of insertion: 3 cm). At this point, a mixture of 3 mL of saline and 0.5 mL of air was injected through the catheter, but no hyperechoic flash could be confirmed in the paravertebral space. Immediately before the start of surgery, 7 mL of 0.375 % ropivacaine (1.85 mg/kg) was administered in divided doses over 1 min. At this point, the catheter was aspirated, but there was no blood regurgitation. Approximately two minutes after the start of ropivacaine administration, the electrocardiographic waveform showed asystole. Immediately, chest compression was started, and adrenaline (0.1 mg) was intravenously administered, and a pulse was established after approximately 2 min. Under a tentative diagnosis of intoxication with the local anesthetic, blood was collected through an arterial line to measure its plasma concentration (6 min after ropivacaine administration), and 20 mL of 20 % lipid emulsion was administered (7 min after ropivacaine administration). As the effect of single-dose administration of adrenaline gradually decreased over time reducing the blood pressure (38/20 mmHg) and heart rate (54 beats per minute), the continuous administration of catecholamine was considered necessary. After a catheter for transesophageal pacing was inserted into the esophagus, central venous catheterization was performed. The continuous administration of adrenaline (0.03 μg/kg/min), dobutamine (5 μg/kg/min), and dopamine (5 μg/kg/min) was started. After the hemodynamic parameters became stable, we decided to proceed to the surgery because we believed that pacemaker implantation would also treat the bradycardia caused by ropivacaine. The surgery was done uneventfully. When the catheter was examined after surgery, blood into the catheter (about 12 cm) was observed. Since the blood had coagulated, the catheter was removed.

The patient was transferred to the intensive care unit (ICU) while being intubated. Dexmedetomidine (0.4 μg/kg/h) and fentanyl (0.5 μg/kg/h) were administered to obtain sedation and analgesia, and tapered gradually. The patient was extubated 2 days and discharged 13 days after the surgery without any neurological abnormalities. Subsequently, the results of examination showed that the plasma concentration of ropivacaine was 5.2 μg/mL.

## Discussion

This is the first report on cardiac arrest resulting from TPVB-related intoxication with a local anesthetic. Ropivacaine administered through a catheter may have flowed into circulation, inducing intoxication resulting in cardiac arrest for the following reasons: 1) cardiac arrest occurred immediately after ropivacaine administration, 2) blood regurgitation into the catheter was found after the completion of surgery, and 3) the results of later examination showed that the plasma concentration of ropivacaine was high. At the same time, in the present case, the influence of sympathetic blockade must be considered as a cause of cardiac arrest. A catheter for TPVB was inserted through the 5th intercostal space, which is adjacent to the cardiac sympathetic branch. Considering the fact that the patient had SSS, it is not surprising that sympathetic blockade by ropivacaine administered through a catheter may lead to cardiac arrest. However, there have been no case reports of cardiac arrest or bradycardia after drug administration for TPVB. Lönnqvist et al. [3] reported complications related to paravertebral block in adults and children: 3.8 % of adults and 4.2 % of children had vascular puncture, and 5 % of adults and none in children had hypotension. Neither bradycardia nor cardiac arrest was reported. Regarding epidural anesthesia, there is a case report in which SSS, which had not been indicated, became manifest after the administration of a local anesthetic [4]. However, this did not lead to cardiac arrest. Based on these findings, sympathetic blockade-related bradycardia does not seem to contribute to cardiac arrest after TPVB, as demonstrated in the present case.

Although few prospective studies have investigated the pharmacokinetics or systemic toxicity of ropivacaine, ropivacaine toxicity in adults and bupivacaine toxicity in children have been reported. Knudsen et al. [5] reported that the arterial plasma concentration of ropivacaine at the onset of symptoms of ropivacaine-related intoxication was 3.4 to 5.3 μg/mL in adult volunteers. Chazalon et al. [6] reported an adult with sciatic/saphenous nerve block-induced bradycardia, leading to cardiac arrest. They indicated that the plasma concentration of ropivacaine after 70 min was 1.88 μg/mL. Huet et al. [7] reported that the plasma concentration of ropivacaine 55 min after cardiac arrest associated with lumbar plexus block was 5.61 μg/mL. In addition, Gnaho et al. [8] indicated that the plasma concentration of ropivacaine 15 min after ventricular fibrillation associated with sciatic nerve block was 2.48 μg/mL. Thus, the plasma concentration of ropivacaine reportedly ranged from 1.88 to 5.61 μg/mL although the interval from cardiac arrest until blood collection varied. Agarwal [9] and McCloskey [10] reported several cases of bupivacaine systemic toxicity secondary to continuous infusion (caudal epidural and intrapleural) in children. The blood levels at the time of seizures (four cases, 3 to 9-years-old) ranged from 5.4 to 10.2 μg/mL. The blood levels in one 3.89 kg-neonate with the initial episode of ventricular tachycardia was 5.6 μg/mL. Although they have reported bupivacaine toxicity, higher plasma concentrations of ropivacaine may be necessary to induce systemic toxicity because plasma concentration of ropivacaine was higher than that of bupivacaine when the symptoms were elicited [5]. In our case, the plasma concentration of ropivacaine was 5.2 μg/mL. As the blood was collected 6 min after cardiac arrest, the concentration at the time of cardiac arrest may have been higher. Therefore, our patient may also have had a high enough blood ropivacaine concentration to cause cardiac arrest.

To treat cardiac arrest due to intoxication with a local anesthetic, adrenaline and lipid emulsion are used. In our case, a single-dose administration of adrenaline immediately after cardiac arrest successfully resumed the pulse. After a bolus administration of lipid emulsion, because the heart rate was maintained by adrenaline, the administration of lipid emulsion was not continued. However, both the heart rate and blood pressure were decreasing, requiring transesophageal pacing and the continuous administration of adrenaline, the continuous administration of lipid emulsion may also have been necessary.

Lastly, we discuss preventive measures of intravascular administration in TPVB. There are several TPVB-specific and general measures to prevent intravascular administration. The first TPVB-specific measure is to confirm a hyperechoic flash in the paravertebral space. Yoshida et al. [11] confirmed that the catheter tip was located in the paravertebral space by injecting a mixture of 3 mL of saline and 0.5 mL of air through a catheter and confirming a hyperechoic flash in the paravertebral space. We did not observe hyperechoic flash, but continued the procedure because we believed the lack of hyperechoic flash was possibly due to technical problems, such as inappropriate direction of the probe-tip. We should have checked the flash thoroughly. However, when air was administered several times until a hyperechoic flash is observed, mediastinal emphysema or air emboli may develop in children. The second TPVB-specific method is to confirm the ventral transposition of the pleura using ultrasonography while drug administration through a catheter [2]. In our patient, saline was infused not through a catheter, but through a Tuohy needle, to confirm the ventral transposition of the pleura. The catheter was inserted 3 cm from the needle tip, but we could have inserted less considering the size of her body. Thus, we cannot rule out the possibility of intravascular migration of the catheter tip. We could have confirmed using ultrasonography if the drug reached the paravertebral space by administering the drug through the catheter. However, this method has a limitation: confirmation is not possible immediately prior to the operation when ultrasonography is not available. To avoid intravascular administration, there are also general measures to be taken. Firstly, there is a method to confirm blood regurgitation through catheter aspiration, but when an excessive negative pressure is applied, no blood regurgitation may occur, resulting a false-negative reaction [8]. We also confirmed blood regurgitation by aspirating the catheter, but found no blood regurgitation. Therefore, we cannot rule out the possibility that the negative pressure was excessive. Secondly, a local anesthetic containing adrenaline is used as a test dose. When adrenaline enters blood circulation, tachycardia, an increase in blood pressure and ECG changes, such as T-wave amplitude and ST segment changes, are observed [12]. However, children under general anesthesia may be less sensitive to adrenaline, leading to false-negative reactions in some cases. Furthermore, when a patient is tachycardic like our patient, adrenaline must be carefully administered, considering the influence of the deteriorated tachycardia on the hemodynamics. Thirdly, it is also important to administer drug slowly. When administered in divided doses, the onset of systemic reactions may be delayed by 60 to 90 s [12]. For administration in divided doses, it is also necessary to administer the subsequent dose after a sufficient period of observation.

## Conclusions

We encountered cardiac arrest which occurred after ropivacaine administration for TPVB in a 6-year-old female. This was possibly due to the intravascular administration of ropivacaine. For TPVB, it is important to combine several measures to prevent intravascular administration and administer drugs carefully.

## Consent

Written informed consent was obtained from the parents of the patient for publication of this Case report and any accompanying images. A copy of the written consent is available for review by the Editor-in-Chief of this journal.

## Abbreviations

TPVB: Thoracic paravertebral block
SSS: Sick sinus syndrome
ICU: Intensive care unit
